# Understanding Perceptions and Practices for Designing an Appropriate Community-Based Kangaroo Mother Care Implementation Package: Qualitative Exploratory Study

**DOI:** 10.2196/30663

**Published:** 2022-01-07

**Authors:** Shabina Ariff, Ikram Maznani, Maria Bhura, Zahid Memon, Tayyaba Arshad, Tariq Ahmed Samejo, Shujaat Zaidi, Muhammad Umer, Imran Ahmed, Muhammad Atif Habib, Sajid Bashir Soofi, Zulfiqar A Bhutta

**Affiliations:** 1 Center of Excellence in Women & Child Health Aga Khan University Karachi Pakistan

**Keywords:** kangaroo mother care, low birth weight, neonatal mortality formative research, Pakistan, newborn care

## Abstract

**Background:**

Low birth weight (LBW) is a common outcome of preterm birth, which increases the risk of an infant’s morbidity and mortality. Approximately 20 million infants are born with LBW globally per year. Since a significant number of births in Pakistan take place at home, it is important to focus on the use of kangaroo mother care (KMC), the practice of skin-to-skin contact, in communities to prevent neonatal mortality and morbidity.

**Objective:**

We employed a formative research approach to understand the context of communities and facilities with regard to neonatal care and KMC practice. The broader aims were to inform the design and delivery of culturally appropriate platforms to introduce KMC in communities, and develop effective recruitment and retention strategies of KMC in rural areas of the Dadu district in the Sindh province of Pakistan.

**Methods:**

We conducted focus group discussions, in-depth interviews, and key informant interviews with families of LBW babies, community members, health care providers, and hospital administrators to identify barriers, enablers, and a knowledge base for KMC interventions.

**Results:**

Newborn care practices in communities were found to be suboptimal. The community was generally unaware of the KMC intervention for the care of LBW babies. However, facility health care providers, the community, and family members were willing to provide KMC to improve outcomes. We found significant support from the community members and health care providers for KMC practices. Mothers were also ready to provide intermittent KMC. The administrative staff at the hospitals accepted the introduction of KMC practices for LBW babies.

**Conclusions:**

KMC as a method of treating LBW babies is widely accepted in the community. This formative research provides strategically valuable information that will be helpful for developing effective implementation strategies by identifying common community practices for LBW babies, along with identifying the barriers and enablers to KMC practice.

## Introduction

The neonatal period (first 4 weeks of life) is the most vulnerable time for child survival. Approximately 2.4 million infants die within the neonatal period globally every year, with most deaths occurring in low-and middle-income countries (LMICs) [1,2]. The leading cause of deaths in neonates is attributed to complications that arise from preterm birth [3]. Preterm birth has long-term effects on growth and neurodevelopment functioning, including an increased risk of cerebral palsy, impaired learning and hearing, and visual disorders [4]. Furthermore, preterm birth is associated with high economic and social costs due to the educational and medical needs of the neonate [3]. Low birth weight (LBW), defined as a birth weight<2500 g, is a common outcome of preterm birth, which increases the risk of an infant’s morbidity and mortality [5]. The prevalence of LBW infants in developing countries (16.5%) is more than double that in developed countries (7%) [6].

Currently, Pakistan’s neonatal mortality rate lies at about 44 deaths per 1000 live births [2]. The prevalence of LBW reported in various studies in Pakistan is in the range of 19%-30% [5]. Factors associated with LBW include the high prevalence of home-based deliveries in the absence of skilled birth attendants. Approximately one-third (34%) of all births in Pakistan take place at home, with a higher percentage of home-based deliveries in rural areas (41%); thus, it is important to focus on appropriate practices for LBW care in communities and rural settings. [7].

A newborn, particularly one born preterm or with LBW, is vulnerable and requires critical care. This challenge largely looms in LMICs where the rates of preterm and LBW babies are higher and the resources available for their care are scarce. Many health facilities are characterized by a lack of staff availability and training, along with ill-functioning equipment with nonexistent neonatal care units, which ultimately result in higher neonatal mortality [8,9]. Furthermore, essential neonatal care for small babies requires incubators and skilled personnel, which are unaffordable and inaccessible by most of the population in underdeveloped regions [10-12].

In response, the kangaroo mother care (KMC) method for the care of preterm and LBW babies was developed over 30 years ago, which is practiced through continuous and long-term skin-to-skin contact between the baby and an adult [13]. KMC is a gentle and effective method of caring for preterm and LBW babies, designed to increase the contact between parents and their infant, and has shown a 40% improvement in survival of preterm infants [14,15]. Advantages of KMC include the reduced risk of hypothermia [16]; improved rate and duration of breastfeeding [17]; early initiation of breastfeeding [18]; sustenance of the infant’s physiological parameters, including respiratory rate and heart rate [19]; and improved mother-infant attachment and bonding [20]. Furthermore, KMC may improve an infant’s head circumference growth and contribute to weight gain [21]. KMC also reduces parental distress related to their infant’s well-being due to constant attachment [22].

The clinical efficacy and health benefits of KMC have been established in multiple settings of developed and developing countries. However, despite the evidence of its benefits, the uptake and implementation of KMC are limited in many developing countries, including Pakistan. Therefore, we conducted a formative assessment to evaluate the barriers and enablers associated with KMC uptake among families, communities, and health services related to newborn care practices in a rural area of Pakistan. These perspectives can help to identify culturally acceptable ways of implementing KMC in the health facilities and communities of this region.

## Methods

### Study Design, Participants, and Setting

A qualitative exploratory study was conducted using focus group discussions (FGDs), key informant interviews (KIIs), and in-depth interviews (IDIs). Study participants included community members (mothers, grandmothers, and fathers), health care providers, lady health workers (LHWs), lady health visitors (LHVs), traditional birth attendants (TBAs), nurses, pediatricians, gynecologists, and *taluka* (subdistrict) hospital administrators. LHWs are first-line community health workers employed by the government to provide maternal newborn child health at the primary-care level and in the community. They provide an essential link between the formal health system and the community. LHVs are a trained cadre that are stationed in the facility and are responsible for conducting deliveries, recognizing high-risk pregnancies, and providing appropriate referrals. They are also trained in immediate newborn care. Mothers were selected for interviews if they had a newborn under 6 months of age and were residents of the project area. LHVs and nurses interviewed were required to have worked in the project area for at least 5 years. For FGDs, family members were required to have a baby in their household with a maximum age of 2 years. The study was conducted in three *talukas* (Nehar, KN Shah, and Johi) of the rural district Dadu in the Sindh province of Pakistan.

### Development of Interview Guidelines

The core research team comprising the principal investigator and coinvestigators developed the interview guidelines. The guidelines were developed in English and translated into the local language. The conceptual framework used for formulating the guidelines was adapted from a KMC formative study conducted in India [23]. Findings from a situation analysis of KMC in Pakistan were also integrated for developing key themes and questions [24]. The guidelines were pretested to check the clarity, accuracy, and flow of questions and probes, and reviewed in a 3-day-long workshop by experts in informative research from Aga Khan University, Pakistan. The experts included anthropologists, study investigators, physicians, and sociologists.

The interview guides were designed to evaluate the knowledge and perception of the community about KMC practices. They contained questions about the community’s understanding of preterm birth complications, the prevailing beliefs and traditional practices regarding newborn care in general, and care of LBW newborns in particular. Questions about existing social support systems for mothers of newborns, the willingness of mothers to provide KMC, support available to mothers practicing KMC, and the willingness of community members to become KMC champions were also included in the guides. The hospital administrators of *taluka* hospitals were also asked about the potential role of health care providers in implementing KMC and suggestions for KMC implementation at the facility level.

### Pilot Testing

Before pilot testing, two training sessions were conducted for the data collectors. One was facilitated by a neonatologist well-versed in KMC. This training involved orientation to the concept of KMC and demonstration of the KMC technique. Participants were educated on various wraps and methods used to hold the baby and establish skin-to-skin contact. A practice session on the KMC position was carried out using a doll. The second training was carried out in a 3-day-long workshop on formative research methods by the core study team.

Following the training, pilot testing was conducted in a village with similar socioeconomic and demographic characteristics as those of the project area. Six mothers, one male community member, and one grandmother participated in the pilot. The results from the pilot were incorporated to modify the study instruments. Moreover, the KMC technique was demonstrated to nine mothers and one grandmother to evaluate the experience of securing the baby in the KMC position with the local KMC wrap.

### Data Collection

Information was collected with the help of six data collectors, including two LHVs and four social scientists with a proven qualitative research background. LHVs are typically the first point of care during the reproductive health period. They have a formal education up to grade 10 and undergo a 2-year training program that comprises 1 year of training in midwifery and 1 year in pediatrics and tropical diseases. LHVs conduct deliveries at the household and facility levels and provide immediate newborn care. The social scientists had master’s degrees in social sciences. All of the data collectors were from the same community and were fluent in the local language. Data were collected between August and November 2017.

Details of qualitative assessments are provided in Table 1. Each FGD comprised 8 to 10 participants and lasted 60 to 90 minutes. KIIs lasted for 35-40 minutes each, which were conducted under the aid of a semistructured topic guide. Additionally, nine IDIs were conducted with mothers, grandmothers, and LHVs/nurses.

**Table 1:** Details of qualitative assessments by community and health services members.

Qualitative assessment type	Community members	Members of health services
Focus group discussions (n=18)	Mothers (n=7), grandmothers (n=4), fathers (n=4), community elder (n=1)	Lady health workers (n=3)
In-depth interviews (n=9)	Mothers (n=2), grandmothers (n=2)	Nurses (n= 1), lady health visitors (n= 4)
Key informant interviews (n=8)	Not applicable	Pediatrician (n=1), gynecologist (n=1), traditional birth attendants/community midwives (n=3), *taluka* hospital administrators (n=3)

Verbal consent was provided by the study participants and recorded on tape. All FGDs, KIIs, and IDIs were conducted in Sindhi, the local language of the area, and were tape-recorded. The recordings were also transcribed in Sindhi. Notes of all sessions were taken by a note-taker in Sindhi.

**Figure 1:**
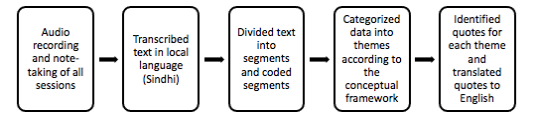
Conceptual framework to asses thematic areas.

A video of KMC being practiced was shown to all participants and their reactions were observed. The video was recorded and developed by a professional firm that was outsourced. A Term of Reference was developed for this purpose. The video was 10 minutes long and was recorded in the native language. The core study team, including the principal investigator and coinvestigators, developed the storyboard for the video. The conceptual model was based on the hypothesis that the adoption of KMC by mothers and by the community at large would be predominantly influenced by factors at different levels: individual and community levels, health care provider, supportive environment, social and cultural factors, and existing practices. The script was originally written in English and then translated into the local language by the study investigators well-versed in both languages. The participating performers in the video were a mixture of professional actors and community members. The principal investigator accompanied the professional crew to the filming and ensured that correct KMC techniques were captured and relevant social aspects incorporated. Figure 1 shows the conceptual framework used to assess thematic areas.

### Data Analysis

The inductive thematic analysis approach was used to analyze the data to determine the themes. First, the data collection team and the investigators familiarized themselves with the data. For the nonnative investigators, the data were transcribed into the English language. Next, the data were coded based on the content to describe the most interesting and important findings. The codes were then classified into common themes. Finally, the themes were reviewed and finalized based on the relevance to answering the research questions.

The process of data collection and analysis is presented in Figure 1. The transcripts were divided into segments, which were then labeled with codes to categorize them and to further develop themes. Transcripts and notes were analyzed on NVivo (Version 11.0). Data were coded according to previously defined assessment areas and thematic contents. The inductive thematic analysis approach was used to analyze the data to determine the themes. First, the data collection team and the investigators familiarized themselves with the data. For the nonnative investigators, the data were transcribed into the English language. Emerging themes and subthemes were identified during this process. Next, the transcripts were divided into segments, which were then labeled with codes according to the themes and subthemes developed in the previous step. The data were further analyzed for main themes and subthemes during coding. The codes were then grouped into common themes. Finally, the themes were reviewed by the investigators and finalized based on the relevance to answering the research questions.

New themes were also identified according to the findings from the transcripts. Since all transcripts were in Sindhi, all data analysis was also performed in Sindhi. The analyzed quotes were then translated to English for reporting.

### Ethical Considerations

The study was approved by the Ethical Review Committee of Aga Khan University (ERC #2020-0321-8561). Informed consent was obtained from all participants before study commencement.

## Results

### Participant Characteristics

We interviewed a total of 184 respondents, including 43 (23.3%) men and 141 (76.6%) women. The respondents included mothers, fathers, women of reproductive age, grandmothers, elderly male members of the community, community village committee members, fathers, and community and facility health care providers (LHWs, LHVs, physicians, and TBAs). The participants ranged in age from 16 to over 50 years. A total of 66 mothers participated in the study. The mean age of the mothers was 31.9 (SD 7.3) years and that of the fathers was 30.3 (SD 9.0) years.

### Theme 1: Community Practices and Perception on Immediate Newborn Care for All Babies and LBW Babies

#### General Practices

Soon after birth, the newborn’s umbilical cord is cut with a blade, and *Surmo*, oil, *Desi Ghee,* spirit, and betamethasone cream are applied to the umbilicus to prevent infections. It is a common belief that newborns are born “impure” and are covered with dirt and substances of the uterus; thus, bathing allows for purification. Once the baby is bathed and is cleared of impurities, the call to prayer can be uttered. Uttering the call to prayer (*Azaan*) in a baby’s ear is a sacred practice in Islam to enable starting one’s life with the name of God: “When babies come out from mothers’ bellies they are covered with impure substance and we wash them first and prepare them for Azaan because we are Muslims.” [Grandmother]

Baby massage is widely reported as an essential component of immediate newborn care in the community. Some other unique practices include applying the saliva of the mother to the infant’s ankles as “protection from evil spirits.” The cultural concept of *chilla* (baby and mother dyad rooming-in) for approximately 40 days is extensively practiced in the community. It is believed that the newborn needs the mother to protect them against evil spirits. Some families even practice keeping sharp objects under the pillow to ward off evil spirits.

When mothers and grandmothers were enquired about the unique issues related to small babies and if they were able to differentiate small babies from normal-size babies, the majority were able to do so and characterized small babies as weak (*kamzor*), those born early (*sattria*), and those with LBW (*ghatwazan*)*.* They were knowledgeable about the special needs of small babies; almost 70% of these respondents stated that such babies require extra care. Two-thirds of the women responded that these babies are slow, it is difficult to feed them, and they become cold very quickly, and therefore they defer the bathing practice in such babies beyond day 1 of life. Approximately half of the mothers and grandmothers stated that the *kamzor* babies are bathed at 2 to 3 days of age, and in the winters such babies’ first bath is delayed to 2 weeks or even more. One-third of the mothers recognized small babies as “sick and unwell” when there is less movement or if the baby is cold (*kamzori*)*:*

If a baby is born ghat wazan waro (kamzor) we give the bath after 2 to 3 days.

Mother

During the winter season, LBW babies are not given a bath and if they become sick doctors tell us not to give bath to the baby till they are well and breastfeeding.

Mother

#### Feeding Practices in LBW Babies

Exclusive breastfeeding is not common across the community, although the mothers and grandmothers were aware of its benefits and understood that breast milk was a nutritious diet, especially for LBW (*ghatwazan*) babies. Both mothers and grandmothers perceived that a small baby must be frequently breastfed. One mother explained, “I used to give my milk to my LBW baby after every 30 minutes unless he would fall asleep.”

When an LBW baby is not feeding well or is unwell, they consult a doctor at a nearby health facility, where they are usually advised to give additional formula milk for extra calories.

We visit doctors for treatment of weak [kamzor] babies and they tell that we must feed them our breast milk and also they tell us to feed formula milk [dabe waro kheer] and medicines and drops for energy [multivitamins].

Mother

The practice of giving goat milk to the baby in addition to breast milk was common, which was usually given in the evening hours*:* “When I gave birth to my baby, I fed him my milk and then I started feeding him goat-milk in the evening.” [Mother]

Regarding colostrum, we found mixed views, where some women were advised by doctors that it is equal to 100 injections (*teeka*) and that it keeps babies healthy, whereas others were advised that colostrum was thick (*kharr*) and dirty and must be discarded: “I had a baby who was born early [*sattria*] and I gave him colostrum [*ghantoo kheer peri khanj*], the doctors say that it is good for the health of the baby.” [Mother]

In addition, infants were frequently given prelacteal feeds such as cardamom (*nonehaal*) gripe water and (*ghutti*) for small babies to help the digestive system.

#### Community Traditions and Rituals for Keeping the Small Baby Warm

LBW babies are generally wrapped in cotton wool and blankets immediately after birth up to 2 to 3 months of age owing to the fear of hypothermia (*thaand*). It is perceived by the community that wrapping them in warm clothing or cotton promotes their growth and well-being. *Kamzor* babies are given sunbaths to keep them warm*:* “When we have small babies, we keep them in cotton for some time and also wrap them in blankets.” [Grandmother]

LBW babies are considered weak and vulnerable, and oil massage is given less frequently compared to the frequency with a healthy normal-size baby; when it is performed on LBW babies, it is typically done gently with less force*:* “We massage LBW babies very less frequently and do it gently because they are weak [*kamzor*] and cannot bear the massaging.” [Mother]

#### Care-Seeking Practices for LBW Babies: Facility Providers’ Views

Hospital administrators shared that most communities consult TBAs and doctors when their newborn is unwell. Communities also seek care for LBW babies from TBAs and LHWs. Additionally, it is a common practice to seek assistance from neighbors and community elders and from mothers who have delivered an LBW baby in the past. According to physicians, the most common traditional practice prevailing in the community for the prevention of hypothermia in LBW babies is swaddling. The baby is swaddled in cotton or wool and placed near a wooden fire in the winter season. The health care providers considered this practice as unfavorable for exclusive breastfeeding and bonding.

#### Place of Delivery and Feasibility of Direct Skin-to-Skin Contact Immediately After Birth

It is a custom for the mother-in-law or elderly male members of the family to decide the place of birth. Mothers are usually discharged from the hospital within hours of delivery. However, in the case of a Cesarean section, they remain admitted for 24 to 48 hours. When enquired about prolonging the hospital stay to learn and provide KMC, the majority of mothers were willing to do so. However, the facility staff shared some challenges that included unavailability of meals for the attendant accompanying the mother, lack of security, unavailability of clean water, and lack of available washroom facilities. The family members were mindful of the attitude of staff toward patients, which largely determines the decision for early discharge and care-seeking practices. Limited finances also were raised as a major barrier to the hospital stay.

We stay at the hospital according to doctor’s advice; whatever he suggests we follow.

Mother, FGD-6

Whatever days are required we can stay for the sake of the health of our baby, may it be for 6 days or more even unless our baby recovers completely.

Grandmother, FGD-2

### Theme 2: Community Perceptions of KMC and Willingness to Adopt KMC Practice

#### Perspectives of LHVs and LHWs on KMC

Some of the LHVs were aware of the benefits of skin-to-skin contact and had heard about KMC in the training they received from a nongovernmental organization on newborn care. However, they were not confident to practice or teach mothers KMC in the community and facility: “We never practice it because of fear, and we do not know the steps of KMC.” [LHV]

They shared that they had never viewed the steps of KMC administration but after watching the KMC video, they thought this care was easy, practical, and would be beneficial for the growth and well-being of the baby.

The LHWs were not familiar with KMC practices. One of the LHWs said, “I have heard about KMC from my colleagues, but I have never seen it and never received any training.”

#### Physicians’ Knowledge and Perception of KMC and its Feasibility

The gynecologist and the pediatrician interviewed had somewhat better knowledge and understanding of KMC and were supportive of the practice. The general views among various health care practitioners were that KMC was a feasible, easy, and adaptable practice in present social and cultural settings in the community. They stated that the local culture encourages other family members, including the husband, mother-in-law, and sisters-in-law, to share the mother’s responsibility during the *chilla* days, and that this supportive family system would facilitate KMC practices at the household level and improve sustainability: “We see mothers-in-law and husbands coming here to support baby and mothers, family members assist each other.” [Pediatrician]

#### Willingness to Practice KMC and Reactions to the KMC Video and Pictures

The majority of mothers and grandmothers were excited and intrigued while watching the video as they had never heard of or seen such practice. As the video was developed in a similar setting to the project site, they expressed happiness and felt connected to the language and the performers in the video. Many believed that KMC appeared to be an effective method as it would *keep the baby warm* and *close to the mother*. Mothers and grandmothers called this practice “*Chhati laen*” (placing on chest) and agreed that the practice would help enhance bonding between the baby and the mother and also enhance breastfeeding. When enquired about its acceptance, the grandmothers expressed that the KMC method will likely be culturally acceptable.

Yes sister [baji], it is a good way, the baby will get more and more breastmilk and will become healthy.

Mother

It is free treatment; no need to spend money, we can also do it at home when we are free.

Father

Yes, this is a good way, a baby will have enough mother-milk, a baby will feel emotionally good, and will gain weight.

Grandmother

#### Views of Mothers and Grandmothers on the Duration and Feasibility of Practicing KMC

Most mothers agreed to administer KMC for an average of 6-8 hours a day without any support from their husbands and other family members. However, they felt that continuous 24-hour KMC would not be possible. They shared their concerns of “backaches” if they were to hold the baby in this position for long hours, especially soon after delivery. They also felt “unsafe” with the baby (skin-to-skin contact) on their chest while sleeping and were concerned about “suffocating” the baby during sleep. Several mothers reported concerns about difficulties in practicing KMC in the summer when excessive heat and humidity cause a lot of perspiration and make KMC administration uncomfortable for both the baby and mother. Many grandmothers were worried that the baby will have sweat rashes. They also expressed anxiety in carrying the baby in this position with a cloth wrapped around their waist and thought that while performing household chores such as sweeping the floor, animal rearing, and washing dishes, they may drop the baby or that the baby may slip out of the wrap.

However, nearly all mothers were still willing to try and practice KMC as they recognize the benefits of this method for their child’s well-being and good health (*suthto*)*.*

I would like to carry the baby with me when the weather is cold, not sure if I want the baby so close to me all the time.

Mother

It is a nice and easy method, and I can do it for 2 hours in the morning, 2 hours in the evening, and 2 or more hours in the night. But it seems difficult when I am performing household chores such as sweeping and washing clothes.

Mother

Adi, I can do the KMC when I am doing chores such as sewing or cutting vegetables, but it seems difficult doing when I am sweeping.

Mother

Although I am busy all the time, I can help my wife with KMC and hold the baby skin to skin contact for 2 to 3 hours in a day.

Father

#### Challenges and Enablers Perceived by the Community for KMC Practices

As the Dadu district has very hot summers, many mothers stated that providing KMC during the daytime may be difficult, but they could hold the baby in the KMC position during the evening and at night. Several mothers suggested that this challenge could be overcome if they were provided with a loose robe (*cholo*) with a zipper or front buttons. The modified garment would make it more comfortable for practicing KMC.

#### Traditional Community Practice of Chilla and Applicability to KMC Practice

*Chilla* is a traditional practice deeply rooted in the rural areas of the country, especially in the provinces of Sindh and Punjab. *Chilla* delineates “care for a birth-giving-woman” in the community. This is the immediate period after delivery and varies from 15 to 40 days. During this period, the mother and baby dyad remain together to ensure bonding and adequate breastfeeding, and the mother is provided time to rest and recuperate her energy. In the first 7 days, she is not allowed to do any household chores. Subsequently, she can perform small tasks that do not require substantial exertion. She is not allowed to carry heavy objects or do fieldwork. *Chilla* is similar to the practice of *attur* in rural Bangladesh; however, their concepts are unique. *Attur* reflects a state of impurity of a delivering woman, whereas *chilla* is purely a social support system during the postpartum period, where family members and *dais* (TBAs) provide support to the woman within a household setting. Several respondents, including grandmothers, expressed that the period of *chilla* would facilitate KMC practice.

Dai [TBA] comes to wash my clothes and my baby’s clothes and massage my body for some days.

Mother

During chilla, days woman is taken care of, she is provided food and she does not do anything but rest till 21 days.

Grandmother

Adi, we rest for 21 days and can do this [KMC] during that time.

Mother

We interviewed three hospital (*taluka* facility) administrators to understand their knowledge and perception of KMC practice for LBW babies. One of the hospital administrators who was from the local community and was a public health expert was aware of the KMC intervention and its benefits. He was of the view that KMC practices can be introduced in the local setting and that the video on KMC would serve well for the uptake. The remaining administrators were unaware of KMC*:* “The main benefits of KMC include exclusive breastfeeding and prevention of hypothermia.” [Administrator]

### Theme 3: Integral Support Systems in the Community to Foster KMC Practices

#### Family and Community Support for KMC

When enquired as to whether other family members such as the grandmother or the father would help in providing KMC, several fathers stated that they would be willing to provide KMC while the mother attends to household chores and the majority were ready to carry the baby in the KMC position for 1 to 2 hours during the daytime.

The mothers believed that with extended family support they could try to provide skin-to-skin contact for a maximum of 10 hours a day*:* “My sister-in-law, my mother-in-law shall also do KMC to my baby if needed.” [Mother]

The majority of facility staff had never heard of KMC; however, after viewing the video, they were supportive and said that although KMC is a new practice for the community, it is a simple and natural method to keep the baby safe and warm.

The LHWs were confident that there is adequate family support for the mother and newborn to facilitate KMC practice. Elaborating on the various tasks to be shared, they stated that other family members, especially a sister-in-law, can prepare food, wash clothes and utensils, clean floors, attend to other children, and also help in cattle rearing and animal dung collection: “Sisters-in-law, cosisters-in-law, the brother-in-law’s wife, and mothers-in-law can provide support to a KMC-practicing mother by taking care of other household chores.” [LHW]

Community elders play a pivotal role in decision-making at the village level and can function as enablers for KMC uptake. The elders were willing to provide their services for KMC advocacy. They were also happy to organize support groups to facilitate KMC practices in the village.

With the formation of support groups and monitoring committee, one can easily be notified of the birth of LBW baby in the village. This will increase coordination among villagers to provide support for mothers with LBW babies and would be a good deed to do.

Elder, FGD

#### TBAs’ Perception and Support for KMC

The TBAs are an old and trusted cadre of health care providers that provide maternal and newborn care and also deliver social support from this platform to the community. They are trusted members of the rural society, and their advice is taken seriously by community elders and decision-makers. They live at the pregnant woman’s house and provide care to the mother and baby dyad during childbirth and the postnatal period. When enquired about their thoughts on KMC, they were not happy with the concept of KMC and considered it as a risky practice. They thought that the baby may slip and fall and they were therefore reluctant to support its practice. Similarly, neighbors were also unwilling to provide skin-to-skin contact to the newborn; however, they were happy to assist in supporting housework. A mother told us that “*Dai* [TBA] comes to massage my body for few days and she helps me to look after my other children.”

### Pilot Testing of KMC Practice

We carried out a pilot study with 9 mothers to evaluate their experience of providing KMC with a local wrap. We used a soft long cloth of cotton material that the women usually wear for modesty locally named *Bhadhno, Rao/Dupatta*, or *Ajrak*

All mothers and one grandmother practiced KMC using *Rao/Dupatta*/*Ajrak* as the KMC wrap. They were trained in binding the local wrap. They felt comfortable holding the baby in the KMC position in the wrap and shared that their babies were safe and relaxed in the KMC position. The KMC wrap was liked by the mothers, as it was very easy to wear and soft to the skin, and most importantly easily available; they expressed that “we don’t need to buy anything also.”

When enquired of any religious or social taboo, we were informed that there were no religious or cultural barriers to practice KMC if they are adequately covered.

When asked “how they felt” with the baby in direct skin-to-skin contact on the chest, the common response was “I feel happy and peaceful, my baby is on my chest and protected.” However, there were concerns of tightness on wearing a shirt over the wrap. Some suggestions were shared, including the availability of a loose robe (*cholo*) with a wide neck (*galo*) and open front that would make the baby and the mother more comfortable and allow for longer hours in maintaining the KMC position.

With babies on the chest (ie, in KMC position), mothers were asked to lie down and incline on the bed, walk and perform some simple household chores, and share their feelings and experience. Overall, the mothers were comfortable and felt good.

### Enablers and Barriers for KMC Implementation

The enablers and barriers promoting and limiting KMC uptake in communities identified through this formative research are summarized in Textbox 1.

**Enablers and barriers contributing to kangaroo mother care (KMC) implementation.**

**Enablers**

- Family members are willing to provide KMC to low birth weight (LBW) babies.
- There exists a caring social support system within the family for a woman giving birth, particularly during the *“chilla”* period, where the baby and mother dyad room-in and the family members ensure that the mother is rested, provided good nutrition, and her daily chore responsibilities are taken over by close family members.
- Community elders are willing to create support groups to facilitate KMC.
- Health care providers understand the benefits of KMC and are willing to support mothers in practicing it.
- Since families take advice from health care providers on health-related matters, it is beneficial for these providers to support, promote, and advise on KMC.
- There is support from religious leaders for the practice as it facilitates breastfeeding and mother and baby attachment.
- The use of diapers is a usual practice in the community that would facilitate keeping the baby dry and in a KMC position for long hours.
- The mother is willing to provide intermittent KMC with a maximum of 2 hours at a time in KMC position.
- Multigravida women are happy to provide support and counseling to young mothers and show interest to demonstrate and administer KMC to the baby.

**Barriers**

- There is no additional community or traditional birth attendant support to provide KMC.
- The community and health care providers generally are not aware of KMC practice and its benefits.
- There is practically no facility in the district where KMC is presently being practiced.
- Health care providers are scarce, especially pediatricians and obstetricians that can provide comprehensive maternal and newborn care at *taluka* hospitals.
- Immediate bathing practices due to social and cultural beliefs may delay the initiation of KMC and could predispose babies to hypothermia.
- KMC is difficult to practice during the day in the summer months due to extreme weather conditions.
- Women are not confident in moving about and carrying out household chores with the baby in the KMC position and fear that the baby may slip out of the wrap. This was considered a significant challenge by the community.
- Keeping the baby in KMC position during the night while sleeping is considered dangerous for the baby.
- Early recruitment of newborns for KMC from the facility would be difficult as the families are typically discharged within a few hours of delivery.
- There is reluctance to practice 24-hour continuous KMC.
- No one had heard of the animal kangaroo or knew how it held its baby.

## Discussion

### Principal Findings

Implementation of KMC in Pakistan has remained elusive until recently, unlike other LMICs where national guidelines for the practice were published as early as 2014 [25]. Most studies have reported the experience of hospital-based KMC in neonatal units with scarce literature on sustainability at the community level. Our study was carried out to evaluate and understand the community and facility drivers and barriers to the implementation of KMC in the country for establishing a sustainable KMC intervention model. The study included perspectives on the beliefs, traditions, family responses, health care providers’ input, and community support for KMC. In addition, we also assessed care-seeking and how an LBW baby is perceived by the family and community.

We found an overall willingness to practice KMC and implement the practice of skin-to-skin contact with the use of a local wrap (*dupatta/chaddar* that the women adorn for modesty) to secure the baby in the KMC position. Our findings are similar to those of a study carried out by Mazumder et al [26] in rural India, where the majority of mothers and grandmothers were happy to practice KMC despite challenges.

The promising finding of our study was the enthusiasm and agreement to practice KMC by the mothers and grandmothers interviewed. They believed in the benefits of KMC and considered it as a healthy practice that would help the LBW baby grow healthy and strong, and protect them from cold. Comparable receptive behavior to KMC practices has been reported in both the local and regional literature on KMC [27-29].

However, almost all of the women who participated in our study were anxious and reluctant to practice continuous KMC, especially during the night hours. They were comfortable giving 1 to 2-hour sessions and a maximum of 8 hours of KMC in a single day. Although the practice of KMC has existed in several countries, there is no evidence on the optimal duration of KMC practice for positive outcomes. Several studies have used a variable duration of KMC practice to demonstrate the impact on reducing mortality in LBW babies. A community KMC pilot study in Bangladesh reported a reduction in neonatal mortality with only 7 hours of skin-to-skin contact in the first 2 days of life and fewer hours in subsequent days [30]*.* Similarly, a qualitative study performed in Indonesia by Mustikawati et al [27] showed that mothers of LBW babies after discharge from hospital practiced KMC for 3 hours per day, and twice and once a day*.*

We identified several inappropriate newborn care practices in the community that have been reported in other published studies. Such practices include early bathing, prelacteal feeding, delayed breastfeeding, use of formula feeds (especially in LBW babies), and providing suboptimal thermal care [31-33]. Some babies are bathed in a traditional norm using immersive bathing immediately after delivery to prevent a bad odor, whereas others are wiped with a damp cloth as a sacred necessity to offer the call for prayer [34]. These traditional practices may serve as barriers to implementing KMC immediately after birth. Nevertheless, the introduction of KMC immediately after birth may enable the replacement of suboptimal practices. At the same time, a strong community awareness program on safe newborn care practices and community mobilization would be required to implement KMC and sensitize the community to safe newborn care. There is sufficient evidence in the literature highlighting the role of health care providers in engaging with the community, especially during the antenatal and postnatal periods. A pilot study performed in India by Rasaily et al [28] demonstrated the positive impact of community mobilization through trained health workers and information, education, and communication provision during antenatal visits on KMC acceptance and practice.

We found mixed perceptions and practices on colostrum, as some women discarded the colostrum due to its thickness and dirty color, while others fed their babies colostrum knowing its impact on immunity and health. The overall opinion of the community was that breastmilk is nutritious and should be offered, even though exclusive breastfeeding practice is uncommon in the community, even for LBW babies. Several mothers were prescribed formula milk (*ka doud*) by the health care providers in clinics and facilities. Similar findings have been reported in qualitative studies from India, Bangladesh, and Indonesia regarding the use of formula feeds [27,34,35]. In Uganda, despite being aware of exclusive breastfeeding, mothers fed babies additional food, including millet or soy porridge, in the hopes of increasing their weight [36]. They also reported differences in feeding practices for LBW babies born in facilities and at home. LBW babies born at facilities were more likely to be exclusively breastfed, whereas babies born at home were more often offered prelacteal feeds, possibly due to a weak suck reflex [36]. In Bangladesh, neonates were given prelacteal feeds, formula milk, and porridges [34]. To improve health outcomes in neonates, it is imperative that newborns, particularly LBW babies, are exclusively breastfed to reduce morbidities and improve their neurodevelopment outcomes [37].

A systematic review performed by Seidman et al [38] highlighted the barriers and enablers of KMC, which found that the most popular enabler of KMC implementation for the preterm infant was family support and mother-infant attachment. Another study on KMC practices in LBW babies at a tertiary-care hospital reported that KMC facilitated warmth, weight gain, and longer sleep hours, and was acceptable by mothers [39]. Moreover, KMC has been associated with significant reductions in neonatal mortality according to an overview of systematic reviews [40].

We found that the postpartum period could be utilized to provide KMC to an LBW baby. *Atur* is a concept in Bangladesh, which is very similar to *chilla* (rooming-in) in Pakistan. This is a 30-45–day–long period where the mother and baby dyad room-in and rest to recover from delivery exhaustion and to encourage breastfeeding and bonding. In Pakistan, this period could be beneficial for mothers in providing KMC to neonates as close family members and friends carry out household chores for the mother [34].

A multicountry analysis on bottlenecks for KMC implementation in 12 countries in Africa and Asia as part of the Every Newborn Action Plan progress concluded that a weak health workforce was a significant bottleneck in 9 of the 12 countries [24]. According to an unpublished report from our department, many barriers to KMC were found, including disagreements from health care providers in the effectiveness of practicing KMC to prevent hypothermia and sepsis. Pakistan has a staggering rate of malnutrition and infectious diseases, and historically the focus has been on these illnesses over issues such as LBW, hypothermia, and neonatal care. Consequently, KMC has not gained acceptance by the health care providers and community until recently (unpublished data of the first author SA, 2014). Strong support systems for mothers and trained health workers can improve the implementation of KMC [38]. Health workers can educate and increase mothers’ knowledge and confidence to practice KMC. The initiation of KMC immediately after birth in the facility by trained health care providers has resulted in sustainable practices in the community in African countries. Studies in Ghana and Nigeria have reported sustainable KMC practices at home following discharge from the hospital [41,42]. In our study, the LHVs were not knowledgeable about KMC and had never observed KMC in the facility, despite its ease and benefit for the growth and well-being of an LBW baby. However, health care providers deemed KMC to be a feasible, easy, and adaptable practice in present social and cultural settings in the community. Women have support available from their families, which can be utilized to practice KMC.

Most of the research in this field has solely focused on accounts by mothers practicing KMC, since cultural gender roles prevent fathers’ involvement in KMC [38]. Before this formative study, we found an unfavorable perception among health care providers and public health specialists in the country for male involvement in providing skin-to-skin contact (unpublished situation analysis report on KMC, first author SA, 2014). This study and the previous literature highlight a critical role of male members in social dynamics and family power structures. We found that fathers were willing and could play a substantial role in the adoption of KMC in the household and for continued practice.

TBAs also play an important role in the birth of a child. KMC was supported in communities in Bangladesh through the role of *dhoronis*, similar to the TBAs in Pakistan*. Dhoronis* play a significant role during childbirth, from delivering the baby to assisting mothers with caring for the baby and doing household chores, and in facilitating KMC to the newborn [34]. The roles across the two countries were similar for the TBA cadre; however, the TBAs in the Dadu district were not comfortable with the idea of mothers providing KMC. This may be due to their perception that empowering mothers with KMC may reduce their importance in providing care to the baby and mother dyad.

To implement KMC in the future, communities at large must be educated on safe care practices for LBW babies with a special focus on exclusive breastfeeding, bathing, and the benefits of skin-to-skin contact [43]. Family members, including fathers, are willing to provide KMC. This willingness can be applied to ensure collective efforts for KMC implementation and sustainability. A mobilization program will maximize the provision of KMC hours by the mother, father, and grandmother. To do so, the implementation teams must be aware of the dynamics of support systems within and outside the family. One approach to formulating a national program for KMC implementation is identifying and training community members to be local KMC champions [24]*.* The tradition of *chilla* can serve as an ideal window of opportunity to implement KMC with counseling and supervision in our setting.

During the demonstration and practice of KMC wrapping by mothers, we recognized the necessity of close liaison, supervision, and a strong support system within the community. The potential of local KMC champions could be explored for offering a source of continuous support to the mothers. A special focus from the mobilization team is also needed to ward off apprehensions about the KMC wrap among mothers and grandmothers, and the safety it poses for newborns. From local dressing patterns, it is inferred that some modification in the form of loose shirts with broad necklines and open fronts may be required. Moreover, establishing newborn inpatient care services at the *taluka* hospitals is required to institute KMC at the facility level.

### Conclusion

The practice of KMC is widely accepted in communities given that it aligns with culturally suited enablers. Families and community elders, including male members, are willing to assist, encourage, and facilitate KMC. Although the adoption of KMC can be facilitated, it would require assistance and counseling from health care providers and community health workers. The government needs to invest in improving newborn care services at hospitals to encourage facility-based KMC.

## Abbreviations

FGD: focus group discussion
IDI: in-depth interview
KII: key informant interview
KMC: kangaroo mother care
LBW: low birth weight
LHV: lady health visitor
LHW: lady health worker
LMIC: low- and middle-income country
TBA: traditional birth attendant
